# Quality of life and long-term outcomes after hospitalization for COVID-19: Protocol for a prospective cohort study (Coalition VII)

**DOI:** 10.5935/0103-507X.20210003

**Published:** 2021

**Authors:** Regis Goulart Rosa, Caroline Cabral Robinson, Viviane Cordeiro Veiga, Alexandre Biasi Cavalcanti, Luciano César Pontes de Azevedo, Flávia Ribeiro Machado, Otavio Berwanger, Álvaro Avezum, Renato Delascio Lopes, Thiago Costa Lisboa, Cassiano Teixeira, Fernando Godinho Zampieri, Bruno Martins Tomazini, Letícia Kawano-Dourado, Daniel Schneider, Denise de Souza, Rosa da Rosa Minho dos Santos, Sabrina Souza da Silva, Geraldine Trott, Bruna dos Passos Gimenes, Ana Paula de Souza, Bruna Machado Barroso, Lauren Sezerá Costa, Liége Gregoletto Brognoli, Melissa Pezzetti Pelliccioli, Nicole dos Santos Studier, Raíne Fogliati de Carli Schardosim, Tainá Aparecida Haubert, Victoria Emanuele Lobo Pallaoro, Debora Mariani de Oliveira, Pedro Isaacsson Velho, Gregory Saraiva Medeiros, Marcelo Basso Gazzana, Alexandre Prehn Zavascki, Paulo Márcio Pitrez, Roselaine Pinheiro de Oliveira, Carisi Anne Polanczyk, Luiz Antônio Nasi, Luciano Serpa Hammes, Maicon Falavigna

**Affiliations:** 1 Hospital Moinhos de Vento - Porto Alegre (RS), Brazil.; 2 Brazilian Research in Intensive Care Network (BRICNet) - São Paulo (SP), Brazil.; 3 BP - A Beneficência Portuguesa de São Paulo - São Paulo (SP), Brazil.; 4 Research Institute, HCor-Hospital do Coração - São Paulo (SP), Brazil.; 5 Research and Education Institute, Hospital Sírio-Libanês - São Paulo (SP), Brazil.; 6 Department of Anesthesiology, Pain and Intensive Care, Universidade Federal de São Paulo - São Paulo (SP), Brazil.; 7 Academic Research Organization, Hospital Israelita Albert Einstein - São Paulo (SP), Brazil.; 8 International Research Center, Hospital Alemão Oswaldo Cruz - São Paulo (SP), Brazil.; 9 Brazilian Clinical Research Institute - São Paulo (SP), Brazil.; 10 Duke Clinical Research Institute, Duke University Medical Center - Durham, NC, United States.

**Keywords:** COVID-19, SARS-CoV-2, Quality of life, Patient-reported outcome measures, COVID-19, SARS-CoV-2, Qualidade de vida, Medidas de resultados relatados pelo paciente

## Abstract

**Introduction:**

The long-term effects caused by COVID-19 are unknown. The present study aims to assess factors associated with health-related quality of life and long-term outcomes among survivors of hospitalization for COVID-19 in Brazil.

**Methods:**

This is a multicenter prospective cohort study nested in five randomized clinical trials designed to assess the effects of specific COVID-19 treatments in over 50 centers in Brazil. Adult survivors of hospitalization due to proven or suspected SARS-CoV-2 infection will be followed-up for a period of 1 year by means of structured telephone interviews. The primary outcome is the 1-year utility score of health-related quality of life assessed by the EuroQol-5D3L. Secondary outcomes include all-cause mortality, major cardiovascular events, rehospitalizations, return to work or study, physical functional status assessed by the Lawton-Brody Instrumental Activities of Daily Living, dyspnea assessed by the modified Medical Research Council dyspnea scale, need for long-term ventilatory support, symptoms of anxiety and depression assessed by the Hospital Anxiety and Depression Scale, symptoms of posttraumatic stress disorder assessed by the Impact of Event Scale-Revised, and self-rated health assessed by the EuroQol-5D3L Visual Analog Scale. Generalized estimated equations will be performed to test the association between five sets of variables (1- demographic characteristics, 2- premorbid state of health, 3- characteristics of acute illness, 4- specific COVID-19 treatments received, and 5- time-updated postdischarge variables) and outcomes.

**Ethics and dissemination:**

The study protocol was approved by the Research Ethics Committee of all participant institutions. The results will be disseminated through conferences and peer-reviewed journals.

## Abstract (pt)

**Introdução:**

Os efeitos provocados pela COVID-19 em longo prazo são desconhecidos. O presente estudo tem como objetivo avaliar os fatores associados com a qualidade de vida relacionada à saúde e os desfechos em longo prazo em sobreviventes à hospitalização por COVID-19 no Brasil.

**Métodos:**

Este será um estudo multicêntrico de coorte prospectivo, aninhado em cinco ensaios clínicos randomizados desenhados para avaliar os efeitos dos tratamentos específicos para COVID-19 em mais de 50 centros no Brasil. Pacientes adultos sobreviventes à hospitalização por infecção por SARS-CoV-2 comprovada ou suspeita serão seguidos por um período de 1 ano, por meio de entrevistas telefônicas estruturadas. O desfecho primário é o escore de utilidade para qualidade de vida relacionada à saúde após 1 ano, avaliado segundo o questionário EuroQol-5D3L. Os desfechos secundários incluirão mortalidade por todas as causas, eventos cardiovasculares graves, reospitalizações, retorno ao trabalho ou estudo, condição funcional física avaliada pelo instrumento *Lawton-Brody Instrumental Activities of Daily Living*, dispneia avaliada segundo a escala de dispneia modificada do *Medical Research Council*, necessidade de suporte ventilatório em longo prazo, sintomas de ansiedade e depressão avaliados segundo a Hospital *Anxiety and Depression Scale*, sintomas de transtorno de estresse pós-traumático avaliados pela ferramenta *Impact of Event Scale-Revised* e autoavaliação da condição de saúde, conforme a Escala Visual Analógica do EuroQol-5D3L. Serão utilizadas equações de estimativas generalizada para testar a associação entre cinco conjuntos de variáveis (1 - características demográficas, 2 - condição de saúde pré-morbidade, 3 - características da doença aguda, 4 - terapias específicas para COVID-19 recebidas e 5 - variáveis pós-alta atualizadas) e desfechos.

**Ética e disseminação:**

O protocolo do estudo foi aprovado pelos Comitês de Ética em Pesquisa de todas as instituições participantes. Os resultados serão disseminados por meio de conferências e periódicos revisados por pares.

## INTRODUCTION

Coronavirus disease 2019 (COVID-19) has received increased attention due to its ability to cause severe illness in a considerable proportion of infected patients.^(1,2)^ Approximately 20% of hospitalized COVID-19 patients develop serious complications, including respiratory failure, acute respiratory distress syndrome (ARDS), shock, delirium, and multiple organ dysfunction.^(3-5)^ Additionally, critically ill COVID-19 patients frequently present a high dependence on organ support with prolonged mechanical ventilation and longer lengths of intensive care unit (ICU) and hospital stay.^(6)^ These factors may result in the reduction of health-related quality of life (HRQL) due to critical illness-associated physical, cognitive and mental health disabilities.^(7,8)^ In this context, observational studies with general critical care survivors have shown a higher occurrence of disabilities such as dependence for activities of daily living, cognitive dysfunction, anxiety, depression and posttraumatic stress disorder (PTSD), as well as lower quality of life and long-term survival compared to the general population.^(9-11)^

Although observational studies assessing the impact of COVID-19 on acute and disease-centered outcomes are available,^(12,13)^ data on long-term outcomes are scarce, and this evidence gap may constitute a barrier to understanding the needs of survivors of severe forms of COVID-19.

The primary objective of this study is to assess factors associated with one-year HRQL among adult survivors of COVID-19 hospitalization. Our secondary objective is to assess the occurrence and factors associated with all-cause mortality, major cardiovascular events, rehospitalizations, return to work or study, physical functional status, dyspnea, need for long-term ventilatory support, symptoms of anxiety, symptoms of depression, symptoms of PTSD, and self-rated health at 3, 6, 9 and 12 months.

## METHODS AND ANALYSIS

### Study design

**Figure 1:**
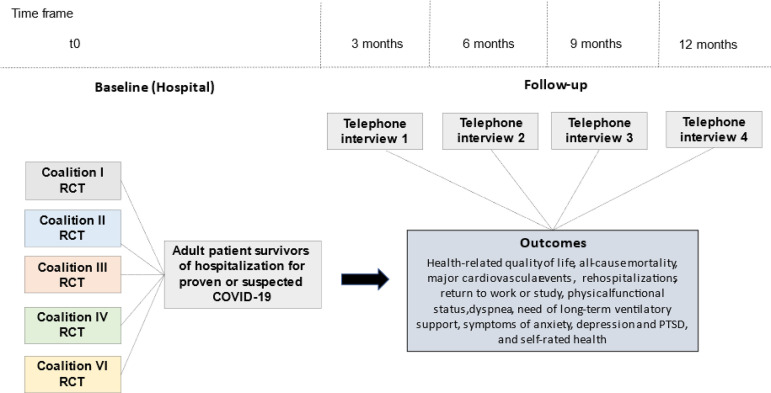
Schedule of enrollment, assessments and follow-up.

The present study is designed as a multicenter prospective cohort study that will enroll patients from five randomized clinical trials that were originally designed to assess the effects of specific COVID-19 treatments in Brazil (Coalition COVID-19 Brazil, Figure 1). Adult patients requiring hospitalization due to proven or suspected severe acute respiratory syndrome coronavirus 2 (SARS-CoV-2) infection will be followed through structured and centralized telephone interviews performed at 3, 6, 9 and 12 months after enrollment. This study protocol was registered at Clinicaltrials.gov (registration number: NCT04376658).

RCT - randomized clinical trial; PTSD - posttraumatic stress disorder.

### Participant eligibility

Patients aged 18 years or older, requiring hospitalization due to proven or suspected SARS-CoV-2 infection and meeting eligibility criteria for Coalition I (NCT04322123),^(14)^ Coalition II (NCT04321278),^(15)^ Coalition III (Codex; NCT04327401),^(16,17)^ Coalition IV (Action; NCT04394377) and Coalition VI (Tocibras; NCT04403685)^(18)^ randomized clinical trials (Table 1) will be enrolled. Patients with a positive polymerase chain reaction result for SARS-CoV-2 will be considered as proven cases. Suspected cases will be defined according to the following factors included in the Brazilian Ministry of Health definition: presence of fever and at least one respiratory sign or symptom (dry or productive cough, shortness of breath, nasal or conjunctival congestion, difficulty swallowing, sore throat, runny nose, oxygen saturation < 95%, signs of cyanosis, rhinorrhea, intercostal circulation and dyspnea) and patients from an endemic region, traveling from an endemic region in the last 14 days, or in contact with a suspected or confirmed case in the last 14 days.^(19)^

**Table 1:** Coalition COVID-19 Brazil randomized clinical trials

Study name (registry)	Population	Interventions	Actual or estimat-ed number of participants
Coalition I (NCT04322123)	Adult hospitalized patients with proven or suspected SARS-CoV-2 infection Needing either no oxygen or a maximum of 4L/minute of supplemental oxygen 14 or fewer days since symptom onset	Arm 1: HCQ Arm 2: HCQ + AZM Arm 3: SOC	667 (actual)
Coalition II (NCT04321278)	Adult hospitalized patients with proven or suspected SARS-CoV-2 infection At least one of the following severity criteria: use of oxygen supplementation of more than 4L/minute flow, use of high-flow nasal cannula, use of noninvasive positive-pressure ventilation, or use of mechanical ventilation 14 or fewer days since symptom onset	Arm 1: AZM + SOC Arm 2: SOC	447 (actual)
Coalition III (NCT04327401)	Adult hospitalized patients with proven or suspected SARS-CoV-2 infection Receiving mechanical ventilation within 48 hours of meeting criteria for moderate to severe ARDS according to the Berlin definition*	Arm 1: dexamethasone Arm 2: SOC	299 (actual)
Coalition IV (NCT04394377)	Adult hospitalized patients with confirmed diagnosis of COVID-19 Onset of symptoms leading to hospitalization < 14 days and D-dimer ≥ 3 x the upper limit of normal	Arm 1: full anticoagulation Arm 2: SOC	600 (estimated)
Coalition VI (NCT04403685)	Adult hospitalized patients with confirmed SARS-CoV-2 infection More than 3 days of symptoms related to COVID-19 Computed tomography (or chest X-ray) with COVID-19 alterations Need for oxygen supplementation to keep SpO2 > 93%, or need for mechanical ventilation for less than 24 hours before the randomization At least two of the following inflammatory tests above the cutoff: D-dimer > 1,000ng/mL, C-reactive protein > 5mg/dL, Ferritin > 300mg/dL, Lactate dehydrogenase > upper-level limit	Arm 1: tocilizumabe Arm 2: SOC	150 (estimated)

SARS-CoV-2 - severe acute respiratory syndrome coronavirus 2; HCQ - hydroxychloroquine; AZM - azithromycin; SOC - standard of care; ARDS - acute respiratory distress syndrome; SpO_2_ - oxygen saturation.

* Acute respiratory failure within 1 week of a clinical insult or new worsening respiratory symptoms AND chest image with bilateral opacities - not fully explained by effusions, lobar/lung collapse, or nodules AND respiratory failure not fully explained by cardiac failure or fluid overload AND positive end-expiratory pressure of 5cmH_2_O or more AND a partial pressure of arterial blood oxygen to fraction of inspired oxygen ratio of 200 or less.

Exclusion criteria include death during hospitalization, absence of telephone contact, absence of proxy for patients with communication difficulties (aphasia, cognitive impairment, severe hearing loss, or non-Portuguese speaker), and refusal or withdrawal of agreement to participate.

### Outcomes

#### Primary outcome

The primary outcome is the 1-year utility score of HRQL assessed by the EuroQol-5D3L questionnaire (EQ-5D3L).^(20)^ The EQ-5D3L comprises a descriptive system with five dimensions of health-related quality of life (mobility, self-care, usual activities, pain/discomfort, and anxiety/depression) and a visual analog scale (VAS) of patient self-rated health. The utility score derived from the descriptive system for the Brazilian population ranges from -0.176 (indicating the worst health status; serious problems in all domains) to 1.0 (indicating the best health status; no problems at all).^(20)^ The minimal clinically important difference estimates of EQ-5D3L range from 0.03 to 0.52.^(21)^

#### Secondary outcomes

Secondary outcome measures include one-year all-cause mortality, major cardiovascular events (nonfatal stroke, nonfatal myocardial infarction, and cardiovascular death), rehospitalizations, return to work or study, physical functional status assessed by the Lawton-Brody Instrumental Activities of Daily Living (IADL),^(22)^ dyspnea assessed by the modified Medical Research Council dyspnea scale,^(23)^ need for long-term ventilatory support (oxygen, noninvasive ventilation, or mechanical ventilation), symptoms of anxiety and depression assessed by the Hospital Anxiety and Depression Scale (HADS),^(24)^ symptoms of PTSD assessed by the Impact of Event Scale-Revised (IES-R),^(25)^ and self-rated health assessed by the EQ-5D3L VAS. All secondary outcomes will be assessed at four time points (3, 6, 9 and 12 months after enrollment).

### Associated factors

Five sets of variables will be assessed as potential associated factors for HRQL and secondary outcomes: 1 - demographic characteristics (age and sex), 2 - premorbid health state (comorbidities, physical functional status one month before hospitalization, previous use of medications, such as corticosteroids, angiotensin II receptor blockers or conversion enzyme inhibitors), 3 - characteristics of acute illness (clinical, radiologic and laboratory manifestations of COVID-19, need for ICU admission, need for ventilatory support, need for neuromuscular blockers, need for vasopressor, need for renal replacement therapy, length of ICU stay, length of hospitalization), 4 - specific COVID-19 treatments received (hydroxychloroquine, azithromycin, dexamethasone, and tocilizumab), and 5 - time-updated postdischarge variables (HRQL, physical functional status and symptoms of anxiety, depression, and PTSD).

### Follow-up

The study will begin at the date of enrollment for coalition randomized clinical trials. Patients will be followed using structured telephone interviews performed 3, 6, 9 and 12 months after enrollment with a 30-day window period (15 days before and 15 days after the estimated date). The telephone follow-up calls will be centralized in a single center composed of researchers who have been trained in the use of standardized telephone interviews and who will be blinded to specific COVID-19 treatments. For patients with communication difficulties, the follow-up interviews will be conducted with their proxy. The proxy will be allowed to answer questions related to the following patient outcomes: mortality, rehospitalization, return to work or study, physical functional status, need for long-term ventilatory support, and HRQL. For each telephone follow-up (3, 6, 9, and 12 months), the patient will be classified as follow-up loss after ten attempts of telephone contact without success at different times on several days within the window period.

### Procedures to ensure data quality

The following procedures will be performed to ensure the quality of the data:

1. To ensure standardization of the study procedures, the investigators responsible for data collection at each participating center will receive at least one training session prior to the beginning of recruitment.
2. The investigators at each participating center will have access to the study coordination centers as a means of dispelling doubts and solving potential problems.
3. The data will be entered on printed standardized data collection forms and stored in an electronic data capture system (REDCap, Vanderbilt University, Nashville, TN, USA).^(26)^ To ensure the adequacy of data transcription, routine doublechecking will be performed as data are entered into the electronic data capture system.
4. A data cleaning routine will be applied frequently. The investigators at the participating centers will be contacted in cases of inconsistencies or missing data. This information will also provide feedback regarding the need for retraining.
5. Telephone interviews will be taped and audited to verify consistency in data collection. The audio files will be anonymously stored in a server that meets the same security norms as those used for data in electronic medical records. Access to the files, which is restricted to the study team, will require user identification and a password.

### Sample size

The sample size of the present study will be determined by the number of patients enrolled in the Coalition COVID-19 Brazil randomized clinical trials who were discharged from the hospital. Considering the in-hospital mortality ratio of published coalition studies (3% to 60%)^(14-16)^ and an estimated one-year survival after hospitalization due to community-acquired pneumonia of 66%,^(27)^ the present study will include approximately 1,000 participants. This sample size will allow a power of 80% to detect a difference of 0.05 utilities (within the range of minimally clinically important difference; 0.03 to 0.52)^(21)^ among two groups of equal sizes at 12 months, with a standard deviation of 0.28 for utility values (estimated based on a previous publication)^(10)^ and an alpha level of 0.05.

### Handling of missing data

The missing values for the variables that compose HADS and IES-R will be imputed, replacing the missing items with the mean of the answered items in the same subscale, if at least half of that subscale has been answered.

### Statistical analysis

We will examine the normality of the data by visual inspection of histograms and by using the Shapiro-Wilk test for normality. At baseline, continuous variables will be expressed as the mean and standard deviation or as the median and interquartile range. Categorical variables will be expressed as counts and percentages.

The adjusted result of the primary outcome (EQ-5D3L utility index) will be summarized for each comparison group using central tendency and dispersion measures, together with the mean or median difference as an effect size measure. All-cause mortality, major cardiovascular events, rehospitalization and return to work or study will be reported as incidence ratios. Physical functional status, degree of dyspnea, need for long-term ventilatory support, symptoms of anxiety, depression or PTSD, and self-rated health will be reported as prevalence ratios using clinically relevant cutoff points. The association between independent variables and outcomes will be assessed using generalized estimated equations accounting for the cluster effect (study of origin) and repeated measures. The variance inflation factor will be used to assess multicollinearity. A statistical significance level of 0.05 will be considered for all comparisons. Since this study is exploratory, we will not adjust analyses of secondary outcomes for multiple comparisons. Sensitivity analyses will be performed considering each coalition study as an independent cohort to check the consistency of the findings. Analyses will be performed with R software (R Development Core Team).^(28)^

## ETHICS AND DISSEMINATION

### Ethics approval and consent to participate

This study will be conducted according to resolution no. 466/12 of the Brazilian National Health Council^(29)^ and the Guidelines for Good Clinical Practice E6(R1).^(30)^ All five randomized clinical trials that compose the present observational study, including their amendments for one-year telephone follow-up to assess quality of life and patient-centered outcomes at 3, 6, 9, and 12 months, were approved by Brazil´s National Ethics Committee (*Conselho Nacional de Ética em Pesquisa* - Conep). Informed Consent will be collected from participants or their proxies at the time of enrollment for one of the five randomized clinical trials that compose this observational study. Participants will be reconsented during the first telephone contact. Enrolled patients or their proxies will have the option to withdraw from participation at any time. Records of participation in this study will be kept confidential and will be accessed in a restricted way only by researchers trained in good clinical practices, who will transfer the clinical information to specific forms (which do not have information that can identify the participants).

### Dissemination

A detailed statistical analysis plan will be made available online. The study results will be submitted for publication regardless of the results after completion. We hope to make the study findings widely available and plan to disseminate our results through conferences and peer-reviewed journals. Due to the pandemic crisis and importance of study findings, we plan to submit preliminary data for publication.

### Data sharing

The authors encourage interested parties to contact the corresponding author with data sharing requests, including for access to additional unpublished data.

## DISCUSSION AND STUDY STATUS

The present study has the potential to clarify the possible impact of COVID-19 on health-related quality of life and long-term outcomes. After hospital discharge, patients affected by serious illnesses may develop physical, cognitive and/or psychiatric disorders that lead to prolonged recovery, higher consumption of healthcare resources, and possible impairment of quality of life.^(31,32)^ In a systematic review of 53 studies, survivors of critical illness consistently reported having a poorer quality of life than healthy controls, even after age and sex adjustments.^(33)^ Although the association between COVID-19 and health-related quality of life and long-term outcomes is plausible, the number of registered studies assessing the association between COVID-19 and long-term patient-centered outcomes is scarce.

The strengths of the present study are its prospective design, the inclusion of a large sample of survivors with severe forms of COVID-19, and the assessment of validated patient-centered outcomes. Potential study limitations include the uncertainty regarding the sample size needed to determine the quality of life and disabilities after hospitalization due to COVID-19, since high rates of mortality and morbidity following severe illness might contribute to losses to follow-up and the inability of the participants to effectively respond to telephone interviews.^(34)^ Additionally, some persistent symptoms of COVID-19, such as anosmia, insomnia, and musculoskeletal complaints, will not be part of the long-term assessment.

The study design and protocol were finalized in March 2020. Coalitions I, II, III and VI have already finished the recruitment of participants. Currently, Coalition IV is enrolling participants. The beginning of telephone follow-ups started in July 2020. We expect to finish the one-year follow-up of all participants between February and April 2022.
